# Shining a spotlight on our reviewers

**DOI:** 10.1039/d1sc90197d

**Published:** 2021-09-24

## Abstract

*Chemical Science* is celebrating Peer Review Week 2021 with the launch of Reviewer Spotlights, a new way to highlight the hard work of our reviewers, and encourage further diversity in our reviewer community.
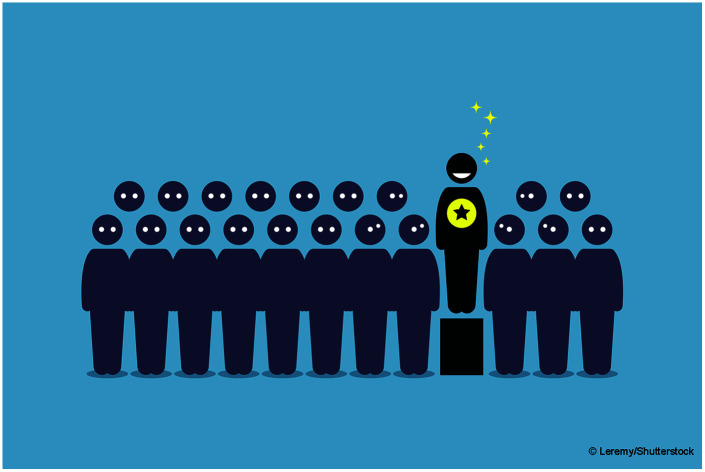

At *Chemical Science*, we recognize the many and varied contributions our reviewer community make to ensure the quality of the research published in the journal is as high as possible.

We also know that a fair and unbiased review system relies on having input from a diverse range of reviewers, whether this is country of origin, gender, ethnicity, career-stage, or affiliated institution, in addition to having the relevant and sufficiently experienced subject expertise.

We want our reviewers to reflect the diversity of our community and are actively working to improve this.

To enable us to improve the diversity of our reviewers, it is important to start by understanding the current situation. As a first step, we have looked at where our current group of reviewers for *Chemical Science* are based, and how this compares with our authorship. This is a relatively easy first step, as this geograhical data is collected as part of the peer-review process and, in the case of the authorship, openly available. We have also started asking our reviewers and authors to voluntarily provide us with data on gender so we may analyse this in a similar way in the future. The other characteristics mentioned above are harder to capture, however we are currently exploring whether we should be doing this, and how this might be possible.

## Who were the reviewers for *Chemical Science* in 2020?

It won’t be surprising to many that the top five countries that make up our reviewers are the US (27%), China (15%), Germany (9%), UK (9%) and Japan (6%).††Data taken from Scholar One, and is the total number of reviewer recommendations returned for articles submitted in 2020, for initial submissions and resubmissions. Reviewer recommendations on revised manuscripts are not included, as these are likely to be the same reviewer in the majority of cases. This top five is the same as the authorship of the articles that are published in the journal.‡‡Data taken from Clarivate Analytics Web of Science™, for all articles published in 2020 issues. This includes all authors on an article. Data accessed 20th September 2021.

The proportions do however differ, with the biggest discrepancy being that 32% of 2020 papers included an author based in China, whereas the percentage of reports returned from reviewers in China was only 15%. The UK is also under-represented in the reviewing pool, making up 18% of papers published but with just 9% of reviewer reports. The US, however, does a more comparable reviewing load for the journal, making up 27% of reviewer reports received, and an authorship of 31% of our published papers. For all other countries, the percentage of reviewer invitations and authorship are within one or two percent of each other.

This picture may not be that surprising, and this generally follows a pattern seen across peer-review globally. The Global State of Peer Review in 2018,^1^ published by Publons and Clarivate Analytics, show the US dominating peer-review (32.9% of all reviews) compared to 25.4% of publication output, with China contributing less (8.8%) when it comes to reviews, compared to article output (13.8%). This same report however does point to this trend reversing in what it refers to as the ‘hard sciences’. When the volume of reviewer reports is normalized by country, this shows China and India making significantly larger contributions across chemistry. The relatively low percentage of reviewer reports from China for *Chemical Science*, and the fact that the proportion of reviewer reports from India is currently 2%, certainly shows that there is work for us to do here.

## Reviewer Spotlight

An important way to encourage new reviewers into the *Chemical Science* community is to ensure we are suitably recognising those that do a great job for us, and show that a broad and diverse range of researchers are welcomed.

Earlier this year, we published our Outstanding Reviewers for the journal (DOI: 10.1039/D1SC90097H), as selected by the editorial team for their significant contribution in 2020 to *Chemical Science*. Our list includes reviewers who provided a much higher than average number of excellent quality reports. We also recognize that providing a high number of reports is not the only important measure, so we also selected reviewers who provided extraordinarily detailed reports, reviewers who were particularly noted for constructively helping authors to improve their manuscripts, and also reviewers who provided noteworthy and thoughtful adjudicative reports as well.

Building on this, it is our continuing aim to thank the many people who contribute their time and effort in support of *Chemical Science*, and so we are now delighted to introduce our new Reviewer Spotlight feature.

Each month we will highlight reviewers who have provided exceptional support to the journal over the past year. They will be featured in a monthly blog post, and we’ll also be talking about an individual reviewer on Twitter (https://twitter.com/ChemicalScience) and Facebook (https://www.facebook.com/chemical.science.journal/) each week.

We are hoping that this feature will also provide some interesting insight into the review process itself and to demonstrate what new reviewers could potentially gain from being part of the process. There might also be some great hints and tips that these reviewers can share with you each month.

To kick start this in August and September, we are highlighting Sangwoon Yoon, Athina Anastasaki, Jeremiah Gassensmith and Yun Chen on our *Chemical Science* blog.^2^ We asked our reviewers a few questions about what they enjoy about reviewing, and their thoughts on how to provide a useful review. To see their answers, please visit our journal blog or follow us on Facebook or Twitter.

## How can you help with reviewer diversity?

• If you are interested in reviewing for *Chemical Science*, or any RSC journal, we are always looking for fresh opinions and voices to be part of the journal community. Please visit our website to see our criteria on becoming a reviewer, and the next steps on how to contact us.^3^

• As an author for *Chemical Science,* when suggesting reviewers for your article when submitting, do consider nominating reviewers from a diverse range of backgrounds and career-stages.

• As an existing reviewer, if you are unable to review an article for *Chemical Science* when invited, please do take the opportunity to give us suggestions of people you know who would do a great job.

And finally as a reviewer, you also receive 50% off a new Affiliate membership for the RSC, which brings further discounts and career support, as well as the chance to track and gain recognition for your reviewing activity through our partnership with Publons.^4^

As always, we love to hear from you so please do get in touch if you have any questions or comments. We look forward to hearing from you.

 

May Copsey, Executive Editor, and the *Chemical Science* Editorial team

chemicalscience-rsc@rsc.org

## Supplementary Material
